# Genetic divergence of Influenza A(H3N2) amino acid substitutions mark the beginning of the 2016–2017 winter season in Israel

**DOI:** 10.1016/j.jcv.2017.05.020

**Published:** 2017-08

**Authors:** Aharona Glatman-Freedman, Yaron Drori, Sharon Alexandra Beni, Nehemya Friedman, Rakefet Pando, Hanna Sefty, Ilana Tal, John McCauley, Galia Rahav, Nathan Keller, Tamy Shohat, Ella Mendelson, Musa Hindiyeh, Michal Mandelboim

**Affiliations:** a The Israel Center for Disease Control, Israel Ministry of Health, Tel-Hashomer, Ramat Gan, Israel; b Department of Epidemiology and Preventive Medicine, School of Public Health, Sackler Faculty of Medicine, Tel Aviv University, Tel Aviv, Israel; c Departments of Pediatrics and Family and Community Medicine, New York Medical College, Valhalla, New York, USA; d Central Virology Laboratory, Ministry of Health, Chaim Sheba Medical Center, Tel Hashomer, Ramat Gan, Israel; e Division of Infectious Diseases, Sheba Medical Center, Tel Hashomer, Ramat Gan, Israel; f WHO Collaborating Centre for Reference and Research on Influenza, Crick Worldwide Influenza Centre, the Francis Crick Institute, London, United Kingdom; g Department of Internal Medicine, Sackler Faculty of Medicine, Tel-Aviv University, Israel; h Microbiology Laboratory, Chaim Sheba Medical Center, Tel Hashomer, Ramat Gan, Israel; i Ariel University, Ariel, Israel

**Keywords:** Influenza A, H_3_N_2_, Influenza vaccine, Clade 3C.2a1

## Abstract

**Background:**

Influenza vaccine composition is reevaluated each year due to the frequency and accumulation of genetic changes that influenza viruses undergo. The beginning of the 2016–2017 influenza surveillance period in Israel has been marked by the dominance of influenza A(H3N2).

**Objectives:**

To evaluate the type, subtype, genetic evolution and amino acid substitutions of influenza A(H3N2) viruses detected among community patients with influenza-like illness (ILI) and hospitalized patients with respiratory illness in the first weeks of the 2016–2017 influenza season.

**Study design:**

Respiratory samples from community patients with influenza-like illness and from hospitalized patients underwent identification, subtyping and molecular characterization. Hemagglutinin sequences were compared to the vaccine strain, phylogenetic tree was created, and amino acid substitutions were determined.

**Results:**

Influenza A(H3N2) predominated during the early stages of the 2016–2017 influenza season. Noticeably, approximately 20% of community patients and 36% of hospitalized patients, positive for influenza_3_), received the 2016–2017 influenza vaccine. The influenza A(H3N2) viruses demonstrated genetic divergence from the vaccine strain into three separate subgroups within the 3C.2a clade. One resembled the new 3C.2a1 subclade, one resembled the recently proposed 3C.2a2 subclade and the other was not previously described. Diversity was observed within each subgroup, in terms of additional amino acid substitutions.

**Conclusions:**

Characterization of the 2016–2017 A(H3N2) influenza viruses is imperative for determining the future influenza vaccine composition.

## Highlights

• A(H_3_N_2_) dominated the early stages of the 2016–2017 influenza season.
• 36% of hospitalized infected patients received the influenza vaccine.
• Circulating A(H_3_N_2_) viruses were different from the vaccine strain.

## 1 Background

Influenza vaccine composition is evaluated each year due to the frequency and accumulation of genetic changes that influenza viruses undergo. The beginning of the 2016–2017 influenza surveillance period in Israel has been marked by the dominance of influenza A(H3N2) virus infection both among community and hospitalized patients. Influenza A(H3N2) is considered the most common cause of seasonal influenza [1], and has been associated with most influenza-related deaths [2].

Hemagglutinin (HA) amino acid (AA) substitutions are considered to occur more frequently for influenza A(H3N2) than influenza A(H1N1) viruses [3], and vaccine effectiveness (VE) against influenza A(H3N2) has been overall lower than VE against influenza A(H1N1) or against influenza B [4]. Thus, it is of paramount importance to detect AA substitutions in the circulating influenza A(H3N2) viruses relative to the recommended vaccine strain as early in the influenza season as possible. In this work, we analyzed influenza A(H3N2) viruses detected early in the season in respiratory samples of influenza patients that were either vaccinated or not vaccinated with the 2016–2017 influenza vaccine.

## 2 Study design

### 2.1 Sample collection

Respiratory samples were collected from patients hospitalized with respiratory illness at the Chaim Sheba Medical Center, Israel, and from patients with influenza-like illness (ILI) from 26 outpatient sentinel clinics throughout Israel. The latter were collected as a part of the Israel Center for Disease Control (ICDC) surveillance program for respiratory viruses morbidity.

Sheba Medical Center institutional review board (IRB) approval was obtained (Helsinki Number 1967-15-SMC).

### 2.2 Laboratory techniques

Viral genomic RNA was extracted from respiratory samples using the NucliSENS easyMAG (BioMerieux, France). Influenza A, Influenza B and A(H1N1)pdm09 infection was detected by using a panel of real-time reverse transcription-PCR (rRT-PCR), as previously described [5], [6]. For influenza A(H3N2) detection and sequencing specific primers were used as previously described [7].

RT-PCR products were sequenced using ABI PRISM Dye Deoxy Terminator cycle sequencing kit (Life Technology, Foster City, CA, USA). Reaction mixtures were analyzed on Applied Biosystems 3500 sequence analyzer.

Nucleic acid sequences were aligned and compared using the Sequencher^®^ 5.0 program (Gencodes Corporation, Ann Arbor, MI). To infer the evolutionary relationships and the most recent common ancestor (MRCA) for the influenza A(H3N2) virus HA sequences, a Bayesian Markov chain Monte Carlo (MCMC) method was applied using a relaxed molecular clock, as implemented in the BEAST program (version 1.7.5). Trees were visualized and edited with the FigTree program (version 1.4.2) [8]. Selected HA sequences were deposited in GenBank under the following accession number designation: KY435815.1, KY435816.1, KY435817.1, KY435818.1, KY435819.1, KY435820.1, KY435821.1, KY435822.1, KY435823.1, KY435824.1, KY435825.1, KY435826.1 (Table 1).

**Table 1:** Influenza A(H3N2) virus hemagglutinin sequences used in phylogenetic analysis.

Virus isolate	Sequence source	Accession Number	Country	Originating laboratory
A/Texas/50/2012	GISAID EpiFlu	EPI377499	United States	Texas Department of State Health Services-Laboratory, Austin, United States
A/Nevada/34/2015	GISAID EpiFlu	EPI633977	United States	Southern Nevada Public Health Lab United States
A/Bolzano/7/2016	GISAID EpiFlu	EPI773595	Italy	Istituto Superiore di Sanità Roma,Italy
A/Hawaii/54/2016	GISAID EpiFlu	EPI814213	United States	State of Hawaii Department of Health Medical Microbiology Branch, United States
A/Wisconsin/85/2016	GISAID EpiFlu	EPI857055	United States	Wisconsin State Laboratory of Hygiene, Virology Unit, United States
A/Illinois/07/2016	GISAID EpiFlu	EPI752842	United States	Illinois Department of Public Health-Chicago, United States
A/Hong Kong/4801/2014	GISAID EpiFlu	EPI539574	China	Government Virus Unit, Hong Kong (SAR)
A/Nebraska/04/2014	GISAID EpiFlu	EPI520356	United States	Nebraska Public Health Lab, United States
A/Switzerland/9715293/2013	GISAID EpiFlu	EPI530687	Switzerland	Hopital Cantonal Universitaire de Geneves, Switzerland
A/Samara/73/2013	GISAID EpiFlu	EPI460558	Russian Federation	WHO National Influenza Centre, Russian Federation
A/New Castele/22/2014	GISAID EpiFlu	EPI543132	Australia	WHO Collaborating Centre for Reference and Research on Influenza, Melbourne, Australia
A/Victoria/361/2011	GISAID EpiFlu	EPI349103	Australia	A Melbourne Pathology, Victoria Pde, Australia
A/Perth/16/2009	GISAID EpiFlu	EP1210071	Australia	WHO Collaborating Centre for Reference and Research on Influenza, Melbourne, Australia
A/Wyoming/3/2003	GISAID EpiFlu	EPI385944	United States	Not available
A/Israel/P451/2015	GISAID EpiFlu	EPI620485	Israel	Central Virology Laboratory (NIC) Israel
A/Israel/P291/2014	GISAID EpiFlu	EPI563104	Israel	Central Virology Laboratory (NIC) Israel
A/Israel/P659/2015	GISAID EpiFlu	EPI620486	Israel	Central Virology Laboratory (NIC) Israel
A/Israel/P151/2014	GISAID EpiFlu	EPI620489	Israel	Central Virology Laboratory (NIC) Israel
A/Israel/P687/2015	GISAID EpiFlu	EPI620488	Israel	Central Virology Laboratory (NIC) Israel
A/Israel/17/2013	GISAID EpiFlu	EPI426073	Israel	Central Virology Laboratory (NIC) Israel
A/Israel/Z1225/2014	GISAID EpiFlu	EPI539814	Israel	Central Virology Laboratory (NIC) Israel
A/Israel/32/2013	GISAID EpiFlu	EPI505186	Israel	Central Virology Laboratory (NIC) Israel
A/Israel/Z774/2014	GISAID EpiFlu	EPI516911	Israel	Central Virology Laboratory (NIC) Israel
A/Israel/19/2013	GISAID EpiFlu	EPI426075	Israel	Central Virology Laboratory (NIC) Israel
A/Israel/Z125/2013	GISAID EpiFlu	EPI515191	Israel	Central Virology Laboratory (NIC) Israel
A/Israel/35/2011	GISAID EpiFlu	EPI354187	Israel	Central Virology Laboratory (NIC) Israel
A/Israel/18/2011	GISAID EpiFlu	EPI319254	Israel	Central Virology Laboratory (NIC) Israel
A/Israel/20/2013	GISAID EpiFlu	EPI426077	Israel	Central Virology Laboratory (NIC) Israel
A/Israel/4/2011	GISAID EpiFlu	EPI319256	Israel	Central Virology Laboratory (NIC) Israel
A/Israel/R83/2016	GenBank	KY435815	Israel	Central Virology Laboratory (NIC) Israel
A/Israel/R147/2016	GenBank	KY435816	Israel	Central Virology Laboratory (NIC) Israel
A/Israel/R199/2016	GenBank	KY435817	Israel	Central Virology Laboratory (NIC) Israel
A/Israel/R245/2016	GenBank	KY435818	Israel	Central Virology Laboratory (NIC) Israel
A/Israel/R246/2016	GenBank	KY435819	Israel	Central Virology Laboratory (NIC) Israel
A/Israel/R248/2016	GenBank	KY435820	Israel	Central Virology Laboratory (NIC) Israel
A/Israel/R250/2016	GenBank	KY435821	Israel	Central Virology Laboratory (NIC) Israel
A/Israel/R255/2016	GenBank	KY435822	Israel	Central Virology Laboratory (NIC) Israel
A/Israel/R256/2016	GenBank	KY435823	Israel	Central Virology Laboratory (NIC) Israel
A/Israel/R274/2016	GenBank	KY435824	Israel	Central Virology Laboratory (NIC) Israel
A/Israel/B5990/2016	GenBank	KY435825	Israel	Central Virology Laboratory (NIC) Israel
A/Israel/B6014/2016	GenBank	KY435826	Israel	Central Virology Laboratory (NIC) Israel
A/Israel/B6105/2016	GenBank	KY435827	Israel	Central Virology Laboratory (NIC) Israel

### 2.3 Cell culture and hemagglutination inhibition analysis

Influenza viruses were grown in Madin-Darby canine kidney (MDCK) cells stable transfected with the cDNA of human 2,6-sialtransferase (SIAT1) as previously described [9].

Hemmagglutination assays were performed using fifty microliters of 0.75% guinea pig red blood cells in the presence of 20 nM oseltamivir carboxylate suspended in PBS. For Hemagglutination inhibition analysis (HAI), two-fold serial dilutions of 25 μl antiserum raised against the egg-propagated A/Hong Kong/4801/2014 vaccine virus were prepared in V-shaped 96 well microtiter plates, and an equal volume of four HA units (determined in the Hemmagglutination assays), were added. The assay was performed with guinea pig red blood cells in the presence of 20 nM oseltamivir. The mixture was mixed by shaking the plates on a mechanical vibrator and then incubated at room temperature for 1 h. Agglutination patterns were read after 60 min and the hemagglutination inhibition (HI) titer was defined as the reciprocal of the last dilution of serum that fully inhibited hemagglutination [10].

## 3 Results

Of the 388 samples obtained from community sentinel patients with ILI from October 2nd through December 9th, 2016, 59 (15.2%) were positive for influenza viruses. Of these, 56 (94.9%) were positive for influenza A(H3N2), one (1.7%) for influenza A(H1N1)pdm09 and two (3.4%) for influenza B virus. A total of 11 (20%) individuals who were infected with influenza A(H3N2) received the influenza vaccine 14 days or more prior to developing ILI.

Out of the 1023 samples received from hospitalized patients, 55 (5.4%) were positive for influenza; of these, 53 (96.4%) were positive for influenza A(H3N2), 1 (1.8%) for influenza A(H1N1)pdm09, and 1 (1.8%) for influenza B. Nineteen (36%) of the influenza A(H3N2)-hospitalized patients received the influenza vaccine 21 days or more prior to hospitalization, 13 were not vaccinated and for 21 patients vaccine information was unavailable. All hospitalized patients had underlying chronic diseases: 34% had cardiovascular diseases, 28% pulmonary diseases, 19% malignancy, 15% hypertension, 6% diabetes, 4% neurological diseases, 4% endocrine/metabolic diseases and 4% were pregnant.

Sequence analysis of the HA gene of 18 influenza A(H3N2) viruses (13 from outpatient sentinel clinic samples, and five hospital samples) that were representative of the entire study period was carried out. Table 2 demonstrates three main different groups of AA substitutions as compared with the vaccine A/Hong Kong/4801/2014 (H3N2) cell-propagated virus. Group 1 had the AA substitutions N171K (HA1), I77V (HA2) and G155E (HA2) which define the newly described clade 3C.2a1 [11]. Group 2 had the T131K (HA1) and R261Q (HA1) substitutions, with an additional substitution in codon position 142 (HA1), consisting of either R142K or R142E. Group 3 had the N121K (HA1) and S144K (HA1) substitutions (Table 2).

**Table 2:** Amino acid (AA) substitutions in the HA gene for influenza A(H3N2) viruses from Israel. Colored cells highlight AA substitutions that are common to all viruses that are part of a certain AA substitution group. Dots represent AAs that match the vaccine A(H3N2) virus sequence. Sample numbers are highlighted in color according to influenza vaccination status: vaccinated–yellow, non-vaccinated–pink, unknown–brown. AA glycosylation is marked in bold letters. (For interpretation of the references to color in this table legend, the reader is referred to the web version of this article.)

Ninety four percent (18 of 19) of the viruses had AA substitutions in antigenic site A (Table 2). Specifically, N121K (HA1) occurred in 67% (12 of 18) of the viruses, S144K (HA1) was observed in 56% (10 of 18), and T131K (HA1) occurred in 22% (4 of 18) of the viruses. Thirty three percent (6 of 18) of the viruses had AA substitutions in position 142 (HA1); 3 (16.7%) were R142K, 2 (11%) R142G and 1 (5.5%) was R142E.

Two AA substitutions, N122D and N246Y, resulted in a loss of a potential glycosylation site (Table 2).

**Fig. 1:**
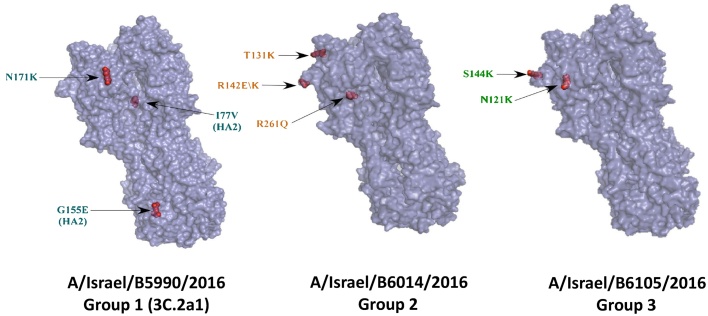
Models of structures of representative influenza A(H3N2) viruses that circulated in Israel in the beginning of the 2016–2017 season. Each structure represents one amino acid combination that circulated in Israel in the beginning of the 2016–2017 influenza season. The arrow labels are colored according to the different groups of aa substitutions, similar to the group colors in Table 2. The 3 groups are indicated.

**Fig. 2:**
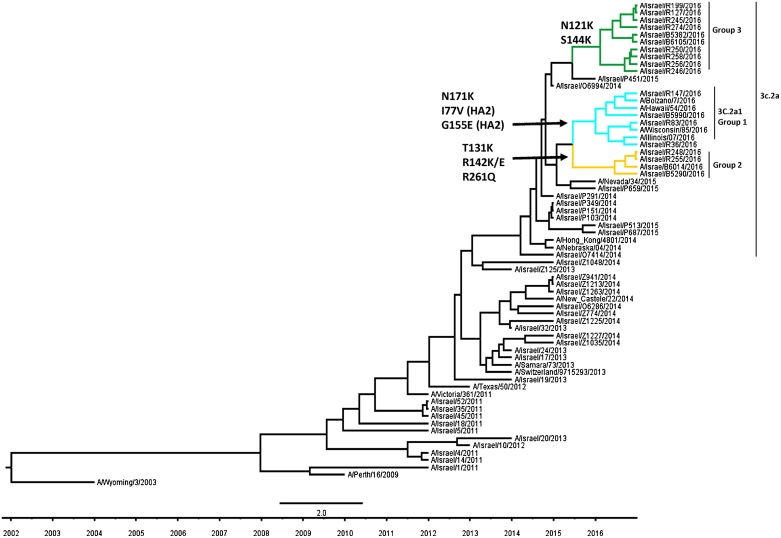
Phylogenetic analysis of hemagglutinin gene sequences of influenza A(H3N2) viruses that circulated in Israel in the beginning of the 2016–2017 season. Analysis was performed with respect to A(H3N2) influenza viruses that circulated in Israel in previous seasons and reference A(H3N2) influenza viruses. These virus sequences were obtained from Global Initiative on Sharing All Influenza Data (GISAID) (Table 1). Colored branches represent different groups of amino acid substitutions, matching the colors of AA substitution groups in Fig. 1. The 3 groups are indicated.

Fig. 1 shows the protein structure of a representative virus from each group of AA substitutions. Phylogenetic analysis of 18 partially sequenced A(H3N2) HA genes (928 base pairs) using the BEAST program showed branching of the influenza A(H3N2) HA sequences (Fig. 2). One branch was related to the previously described 3C.2a1 subclade [11](marked with turquoise branches and designated as group 1) (Fig. 2). A second branch was related to the recently proposed 3C.2a2 subclade [12] (marked with green branches, designated as group 3) (Fig. 2).

To determine if these new clades are associated with an antigenic drift we performed HAI testing (as described in materials and methods), however we were unable to obtain hemagglutination using Guinea pigs red blood cells in the presence of oseltamivir carboxylate. Similarly, 90% of the viruses that were tested by the WHO did not hemagglutinate [14]. Additional influenza viruses from Israel were analyzed for HAI by the WHO Collaborating Centre for Reference and Research on Influenza. Of the six influenza viruses analyzed so far, four could not agglutinate red blood cells at all, and thus could not be analyzed for HAI. The other two influenza viruses that were analyzed by HAI were recognized by an antiserum raised against a cell culture-propagated cultivar of the currently recommended vaccine virus A/Hong Kong/4801/2014 at titers 4-fold lower and 8-fold lower than the titer of the antiserum for the homologous virus. An antiserum raised against the egg-propagated vaccine virus A/Hong Kong/4801/2014 also recognized the two viruses analyzed so far at titers 4-fold or greater lower than the titer of the antiserum for the homologous virus. These results are available in the report of the Vaccine Composition Meeting held in February [13].

## 4 Discussion

Surveillance of influenza viruses at the beginning of the influenza season is of paramount importance for evaluation of current infecting viruses and preparation of the appropriate next season vaccine. Israel is located in the northern hemisphere in western Asia. Influenza A(H3N2) predominated in Israel during the preceding seasons of 2010/2011, 2011/2012, 2013/2014 and 2014/2015, in which it constituted 59.5%, 84.3%, 76.4% and 92.2% of the influenza-positive nasal-throat samples obtained from ILI sentinel patients, respectively [14], [15], [16], [17]. During that period, the influenza A(H3N2) vaccine component was modified several times [18]. Amino acid substitutions in the HA protein are considered to occur more frequently in influenza A(H3N2) than in influenza A(H1N1) [3], [19], [20], and a significant drift was detected during the 2014–2015 season in the northern hemisphere, dominated by the 3C.2a clade [7], [21]. Based on available vaccination data, 20% of ILI sentinel patients and at least 36% of hospitalized influenza A(H3N2)-positive cases became ill in the beginning of the 2016-2017 season despite being vaccinated.

In this regard, during the 2014–2015 influenza season that was dominated by a drifted influenza A(H3N2), approximately 20% of influenza A(H3N2)-positive influenza received the influenza vaccine [7].

Nucleotide sequencing revealed several differences as compared to the 2016-2017 influenza A(H3N2) vaccine strain. These changes demonstrated genetic divergence into three groups of AA substitution combinations. One of these combinations (Group 1) belonged to the newly described clade 3C.2a1 [11] and another (Group 3) was related to the recently proposed clade 3C.2a2. The remaining combination (Group 2) was most recently identified also in Europe [13].

It is interesting to note that influenza A(H3N2) was detected only in 1.7% of positive influenza samples received from outpatient sentinel patients with ILI during the 2015–2016 influenza season in Israel [22]. Thus, lack of a previous year exposure coupled with the mutations observed in the circulating viruses may contribute to the spread and dominance of influenza A(H3N2) during the present season.

Mutations that cause antigenic drift occur most frequently in the gene encoding the HA surface glycoprotein, which constitutes the target of neutralizing antibodies [23]. Influenza A(H3N2) has five neutralizing antigenic sites designated by the letters A to E, with 131 amino acid positions within them associated with antigenic changes [23]. However, seven of these positions (145, 155, 156, 158, 159, 189 and 193), found in antigenic sites A and B, have been more likely to be associated with antigenic changes [23], [24]. None of the AA substitutions detected in our study occurred in these seven positions. However, the HAI results may provide some evidence for a possible antigenic drift. It is interesting to note that recent mid-season influenza A(H3N2) vaccine effectiveness studies estimates ranged mostly between 38% and 43% [25], [26], [27], [28], [29], which are consistent with modest protection overall.

The detection of three clear and different groups of AA substitutions, suggests that continued characterization of influenza A(H3N2) is necessary to see which group will prevail, prior to the decision regarding the future influenza vaccine composition.

In summary, characterization of early-season influenza viruses is important prior to the decision regarding future influenza vaccine composition. Due to the more rapid molecular changes that occur in influenza A(H3N2), detecting these changes is of paramount importance.
